# Case Series of Adverse Pregnancy Outcomes Associated with Oropouche Virus Infection

**DOI:** 10.3390/v17060816

**Published:** 2025-06-05

**Authors:** Daniele Barbosa de Almeida Medeiros, Juarez Antônio Simões Quaresma, Raimunda do Socorro da Silva Azevedo, Ana Cecilia Ribeiro Cruz, Sandro Patroca da Silva, Arnaldo Jorge Martins Filho, Bruno Tardelli Diniz Nunes, Lucas Rafael Santana Pinheiro, Jorge Rodrigues de Sousa, Jannifer Oliveira Chiang, Lívia Carício Martins, Consuelo Silva Oliveira, Ivy Tissuya Essashika Prazeres, Daniele Feitas Henriques, Camille Ferreira Oliveira, Valéria Lima Carvalho, Clarice Neuenschwander Lins Morais, Bartolomeu Acioli-Santos, Keilla Maria Paze Silva, Diego Arruda Falcão, Mayara Matias de Oliveira Marques Costa, Eduardo Augusto Duque Bezerra, Ana Márcia Drechsler Rio, Neijla Cristina Vieira Cardoso, Juliana Carla Serafim da Silva, Simone Gurmão Ramos, Erika Cavalcante Maranhão, José Lancart de Lima, Pedro Fernando da Costa Vasconcelos, Bruno Issao Matos Ishigami, Lívia Medeiros Neves Casseb

**Affiliations:** 1 Department of Arbovirology and Hemorrhagic Fevers, Evandro Chagas Institute, Rodovia BR-316 km 7 s/n-Levilândia, Ananindeua 67030-000, Pará, Brazil; danielemedeiros@iec.gov.br (D.B.d.A.M.); socorroazevedo@iec.gov.br (R.d.S.d.S.A.); anacecilia@iec.gov.br (A.C.R.C.); spatroca@gmail.com (S.P.d.S.); brunonunes@iec.gov.br (B.T.D.N.); lucaspinheiro523@hotmail.com (L.R.S.P.); janniferchiang@iec.gov.br (J.O.C.); liviamartins@iec.gov.br (L.C.M.); consuelooliveira@iec.gov.br (C.S.O.); ivyprazeres@iec.gov.br (I.T.E.P.); danielehenriques@iec.gov.br (D.F.H.); camille.oliveeira@gmail.com (C.F.O.); valeriacarvalho@iec.gov.br (V.L.C.); liviacasseb@iec.gov.br (L.M.N.C.); 2 Department of Entomology, Aggeu Magalhães Institute (IAM), Recife 50740-465, Pernambuco, Brazil; 3 Department of Clinical and Experimental Pathology, Evandro Chagas Institute, Rodovia BR-316 km 7 s/n-Levilândia, Ananindeua 67030-000, Pará, Brazil; juarez@ufpa.br (J.A.S.Q.); arnaldofilho@iec.gov.br (A.J.M.F.); 4 Center for Tropical Medicine, Pará State University, Tv. Perebebuí, 2623-Marco, Belém 66087-662, Pará, Brazil; krekrodrigues@gmail.com; 5 Department of Virology, Aggeu Magalhães Institute (IAM), Recife 50740-465, Pernambuco, Brazil; clarice.lins@fiocruz.br (C.N.L.M.); bartolomeu.santos@fiocruz.br (B.A.-S.); 6 Central Public Health Laboratory of Pernambuco state, R. João Fernandes Viêira, Boa Vista, Recife 50050-215, Pernambuco, Brazil; keillampsilva@gmail.com (K.M.P.S.); diego_arruda2006@hotmail.com (D.A.F.); mayaramatiasoliveira@gmail.com (M.M.d.O.M.C.); 7 Health Department of Pernambuco state, R. Doná Maria Augusta Nogueira, 519-Bongi, Recife 50751-535, Pernambuco, Brazil; eduardo.dbezerra@saude.pe.gov.br (E.A.D.B.); anadrechsler519@gmail.com (A.M.D.R.); sgramos@fmrp.usp.br (S.G.R.); erikacmaranhao@hotmail.com (E.C.M.); jlancart.lima@saude.pe.gov.br (J.L.d.L.); 8 Municipal Health Department of Rio Formoso, R. Barão do Rio Branco, 153, Rio Formoso 55570-000, Pernambuco, Brazil; neijla_cardoso@hotmail.com; 9 Municipal Health Department of Ipojuca, Av. Francisco Alves de Souza 165, Ipojuca 55590-000, Pernambuco, Brazil; serafim.juliana@gmail.com; 10 Department of Pathology, State University of Pará, Travessa Perebebui, 2623, Belém 66087-662, Pará, Brazil

**Keywords:** oropouche virus, vertical transmission, fetal outcomes

## Abstract

The Oropouche virus (OROV) is an arbovirus (Peribunyaviridae: Orthobunyavirus) that traditionally causes febrile outbreaks in Latin America’s Amazon region. Previously, OROV was not associated with severe pregnancy outcomes. During the 2022–2024 outbreak in Brazil, OROV expanded geographically, revealing links to adverse pregnancy outcomes. This study describes six cases with varied fetal outcomes, including miscarriage, antepartum, intrauterine fetal demise (IFD), and normal development, correlating with maternal symptoms but not symptom severity. Vertical transmission was confirmed by detecting OROV through RT-qPCR, ELISA, and immunohistochemistry in fetal tissues. Genome sequencing from an IFD case identified a novel reassortment pattern reported in the 2022–2024 outbreak. Severe encephalomalacia, meningoencephalitis, vascular compromise, and multi-organ damage were evident, underscoring the significant risk OROV poses to fetal development and emphasizing the need for further investigation.

## 1. Introduction

The Oropouche virus (OROV) is an arthropod-borne virus classified into the family *Peribunyaviridae*, genus *Orthobunyavirus*, *Orthobunyavirus oropoucheense* species. The OROV genome consists of three segments of negative-sense, single-stranded RNA, referred to as small (S), medium (M), and large (L). This tri-segmented genome grants OROV the ability of genetic reassortment, meaning the exchange between segments when co-infections occur in nature with other orthobunyaviruses [1]. During epidemics, OROV infection is typically transmitted through midge bites and spreads, sometimes striking both large cities and small communities. The majority of cases result in a self-limited febrile illness known as Oropouche fever, although some individuals may develop central nervous system (CNS) involvement, such as meningitis, encephalitis, and meningoencephalitis [2,3,4].

OROV is primarily found in the Amazon rainforest and is maintained in nature through a complex sylvatic cycle involving sloths and non-human primates as reservoirs and mosquitoes as vectors. Although the specific mosquito species involved in Oropouche sylvatic transmission is not yet known, isolation of OROV from pools of Aedes serratus and Coquillettidia venezuelensis mosquitoes indicates the potential role of these mosquitoes in the transmission cycle in the forest. The urban cycle is simple and direct between humans and Culicoides paraensis midges [5,6,7]. The presence of OROV has also been observed in *Culex quinquefasciatus* urban mosquitoes; however, its capability to transmit the virus has not been proved so far [8]. Vertical transmission has also been highlighted as a pathway for OropFouche transmission, with fetal alterations [9].

Over the past six decades, the occurrence of OROV outbreaks in both peri-urban and urban environments has been reported in several Latin American countries, including Brazil, Peru, Colombia, French Guiana, Trinidad and Tobago, and Panama [4,10,11]. Since the Oropouche reemergence in 2022 in Brazil, which started in the Amazon region [12,13], until the epidemiological week 14 of the 2025 year, the Brazilian Ministry of Health (BMH) reported around 22,380 confirmed cases by RT-qPCR [14]. During this reemergence of OROV, all five regions of Brazil reported cases, including the states of Bahia, Ceará, Espírito Santo, Santa Catarina, Minas Gerais, Rio de Janeiro, Paraná, Piauí, Maranhão, Mato Grosso, Mato Grosso do Sul, and Pernambuco, all of which had not previously reported Oropouche fever cases [15,16,17]. There were at least two confirmed death cases in Brazil during 2024 related to Oropouche fever [14,17,18]. Bolivia, Ecuador, Guyana, Canada, the United States, Barbados, Colombia, Cuba, the Dominican Republic, the Cayman Islands, and Peru also reported OROV circulation. It was the first time that OROV had been detected in some of these countries, such as Cuba [19,20].

In this study, we present molecular, histological, and immunohistochemical analyses of four cases that confirm the association between OROV infection, vertical transmission, and fetal loss.

## 2. Materials and Methods

### 2.1. Study Setting

Following the BMH’s directive to investigate other arboviruses, 10% of negative samples tested for the Dengue (DENV), Zika (ZIKV), and Chikungunya (CHIKV) viruses by RT-qPCR were also tested for OROV, in response to its spread to regions outside the Amazon rainforest [12]. OROV investigation began in the state of Pernambuco in May 2024. Initial cases were identified in the municipalities of Maraial, Rio Formoso, Ipojuca, and Jaqueira, located in the Zona da Mata region. This region is historically and economically significant for the state, characterized by lush vegetation, fertile soil, and mangroves that support extensive sugarcane and banana cultivation. Despite agricultural expansion and habitat fragmentation, the region maintains a rich diversity of flora and fauna. These environmental features, including the proximity to mangroves, contribute to sustaining Culicoides midges, which are key vectors in the transmission of OROV to humans.

Crucially, the presence of Culicoides midges near the residences of the pregnant women under investigation, along with the occurrence of other OROV infection cases in these municipalities, constitutes a significant epidemiological factor that strengthens the association and supports the conclusion of the vertical transmission case investigations.

### 2.2. Ethics Statement

Biological samples from the patients were collected and processed during the 2024 OROV epidemic surveillance in Brazil and analyzed as part of a research study investigating OROV cases, which was submitted to the Research Ethics Committee (CEP) of the IEC and approved (CAAE: 43168321.5.0000.0019). 

### 2.3. Case Reports

**Figure 1:**
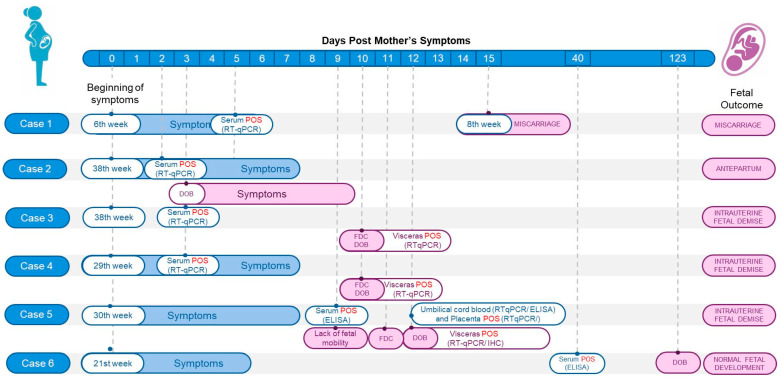
Flowchart illustrating the timeline of six pregnancy cases, starting from the first day of maternal symptom onset. Maternal information is displayed in blue, including gestational age, symptom duration, and laboratory results confirming OROV infection. Pink shows fetal or newborn data, such as confirmation of fetal death by ultrasound (FDC), date of birth (DOB), sample collection dates, laboratory results, and pregnancy outcomes: miscarriage, antepartum, intrauterine fetal demise (IFD), or normal fetal development (NFD). legend: POS = positive result; RT-qPCR = real-time (RT)-PCR; ELISA = enzyme-linked immunosorbent assay; IHC = immunohistochemistry.

Among the OROV-positive cases, four pregnant women were identified: Case 1, which resulted in a miscarriage; Case 2, associated with antepartum OROV infection; Cases 3, 4, and 5, which involved intrauterine fetal demise (IFD); and Case 6, a symptomatic mother without fetal loss. During the investigation, serum samples were collected from the mothers at different times after the onset of symptoms, as well as from the newborns one month after birth. It was not possible to collect samples from the fetus of the miscarriage case. For the IFD cases, samples were collected from umbilical cord blood and tissue fragments of umbilical cord, placenta, and fetal tissues, which were tested using both molecular biology and/or histopathological analyses (Figure 1).

### 2.4. Serological Testing

An in-house immunoassay for IgM capture (IgM-ELISA) was used to specifically detect IgM antibodies against OROV. The antigen was prepared from infected newborn mice serum using the sucrose–acetone method [21]. The IgM-ELISA [22] assay utilized the 6B6C-1 peroxidase-conjugated monoclonal antibody (CDC, Atlanta, GA, USA) and TMB substrate (SeraCare, Milford, MA, USA). Optical density (OD) readings were taken at a wavelength of 450 nm using a spectrophotometer (BioTek Instruments, ELx800, Winooski, VT, USA). Results were categorized as negative when OD < 0.2, undetermined when OD was between 0.2 and 0.3, and positive when OD > 0.3.

### 2.5. Real-Time (RT)-PCR (RT-qPCR)

Total RNA was extracted and purified using the TRIzol™ Plus RNA Purification Kit (Invitrogen, Waltham, MA, USA). Up to 100 mg of tissue from placenta, brain, liver, kidneys, lungs, heart, and spleen was disrupted in 1ml of TRIzol™ reagent with a 5 mm stainless steel bead using a TissueLyser II (Qiagen, Hilden, Germany). The samples were agitated for 2 min at 25 Hz. After adding 0.2 mL of chloroform, samples were homogenized in a vortex and then transferred to phase-maker tubes (Invitrogen, Waltham, MA, USA) and centrifuged for 15 min (12,000× *g* at 4 °C). Next, 600 µL of the aqueous phase containing the RNA was mixed with the same volume of ethanol 75% and purified using the PureLink RNA Mini Kit (Invitrogen, Waltham, MA, USA) according to the manufacturer’s instructions. For serum, RNA was extracted using the QIAmp Viral RNA Mini Kit (Qiagen, Hilden, Germany) following the manufacturer’s recommendations. RT-qPCR was performed using the commercial kit Superscript III Platinum One-Step RT-qPCR System with separate ROX (Invitrogen, Waltham, MA, USA), with a 25 µL reaction volume containing 5 µL of extracted RNA, 300 nM of each primer, 100 nM of probe, and 50 µM of ROX. OROV-specific primers and TaqMan Minor Groove Binding (MGB) probes have been described previously [23]. RT-qPCR reactions were carried out in duplicate in the 7500 Real-Time PCR System (Applied Biosystems, Waltham, MA, USA) using the following cycling conditions: 50 °C for 30 min, 92 °C for 2 min, followed by 45 cycles of 95 °C for 15 s and 60 °C for 1 min. Samples were considered detectable for OROV RNA when the cycle threshold (CT) values obtained were lower than 38 in both replicates. In addition, all clinical samples were simultaneously tested by RT-qPCR against DENV, ZIKV, and CHIKV [24,25,26].

### 2.6. Next-Generation Sequencing

Total RNA extraction from tissue fragments was performed using the TRIzol™ Plus RNA Purification Kit (Invitrogen, Waltham, MA, USA), as described above. RNA quantification was then conducted with the Qubit RNA HS Assay Kit and Qubit 4.0 fluorometer (Thermo Fisher Scientific, Carlsbad, CA, USA). For cDNA synthesis, the SuperScript™ VILO™ MasterMix Kit (Thermo Fisher Scientific, Carlsbad, CA, USA) and the Second Strand cDNA Synthesis Kit (Thermo Fisher Scientific, Carlsbad, CA, USA) were utilized, and the cDNA was subsequently purified using the PureLink^®^ PCR Purification Kit (Thermo Fisher Scientific Baltics UAB, Vilnius, Lithuania). cDNA quantification was carried out with the DNA HS Assay Kit (Illumina, Inc., San Diego, CA, USA) on the Qubit 4.0. Genomic library preparation adhered to the Nextera XT DNA Kit (Illumina, Inc., San Diego, CA, USA) guidelines and was sequenced on the NextSeq 500 platform (Illumina, Inc., San Diego, CA, USA) using paired-end sequencing methodology.

### 2.7. Bioinformatic Analysis

First, the quality of the reads generated during sequencing was evaluated using the Fastp program [27], with the removal of short reads, adapter fragments, and reads with indeterminate bases. Next, the SortMeRNA v.2.1 program [28] was used to remove ribosomal RNA (rRNA). The files generated during the processing step were used for De Novo assembly using SPAdes v3.13.1 [29] and MEGAHIT v1.2.9 [30] programs. The generated contigs were analyzed using DIAMOND [31] with BLASTX, based on the non-redundant viral protein database (nr), and visualized in Megan. Contigs that showed similarity to viral sequences of interest were visualized in the Geneious Prime program 2024 [32].

Phylogenetic inference was performed using nucleotide sequences of various viral strains of OROV from the NCBI database, focusing on protein-coding regions. Multiple sequence alignment (MSA) was conducted with MAFFT v7 [33] and manually inspected using Geneious Prime [32]. The dataset was analyzed to evaluate the phylogenetic signal using the TREE-PUZZLE program [34]. Phylogenetic trees were constructed using the maximum likelihood (ML) method [35] with IQ-TREE2 v2.2.5 [36], with bootstrap testing (1000 replicates) to ensure clustering reliability. Phylogenetic visualization was carried out with FigTree v1.4.4 [37], employing midpoint rooting. The final phylogeny was edited in the Interactive Tree of Life (iTOL) online tool [38]. Nucleotide and amino acid identity matrices were generated using R software [39].

### 2.8. Necropsy, Histopathology, and Immunohistochemistry

The deceased fetuses were sent to the Death Verification Service in Recife, Pernambuco State, where they were evaluated for maceration, autolysis, and other morphological abnormalities. These findings were classified as mild, moderate, or severe. Fetal tissues were carefully collected from the umbilical cord and various organs immediately post-mortem to minimize degradation. Sections of fetal tissues—such as umbilical cord, brain, liver, spleen, heart, lung, and kidney—were stained using hematoxylin and eosin (H&E) [40] to assess general tissue morphology. Immunohistochemistry (IHC) was performed using an adapted streptavidin alkaline phosphatase (SAAP) assay [41] with a polyclonal anti-OROV mouse antibody. The polyclonal anti-OROV antibody used in the IHC assay was prepared at the IEC, using a strain of OROV isolated in cell culture (Vero cells). All clinical samples were simultaneously tested by IHC for DENV, ZIKV, CHIKV, and YFV. Semi-quantitative analysis was conducted to evaluate tissue damage and differentiate between autolytic changes and pathology attributed to the virus. These combinations of analyses composed the ancillary tests to confirm the presence of OROV and distinguish viral-induced changes from autolytic artifacts [42].

## 3. Results

### 3.1. Investigation

**Figure 2:**
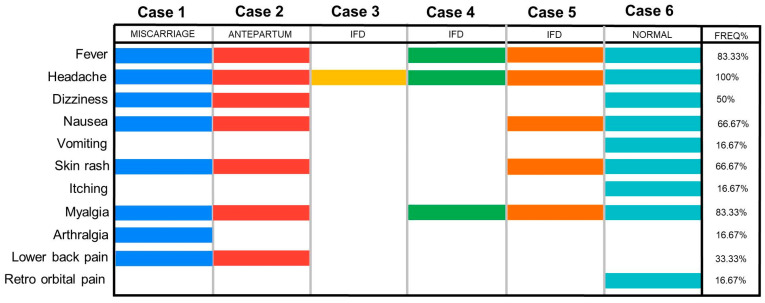
Overview of symptom profiles in pregnant women infected with OROV. Each case is represented by a different color. A wide range of maternal symptoms was observed, regardless of pregnancy outcome. Headache, fever, and myalgia were the most frequently reported symptoms.

In January 2024, the OROV epidemic in Brazil spread through the Amazon region and gradually reached all five regions of the country. Located in the northeast region, the state of Pernambuco detected its first cases of OROV in its history in June 2024 in the municipalities of Maraial, Rio Formoso, and Jaqueira, located in the Zona da Mata, a biome present in peri-urban areas of the state. Among these initial cases, two pregnant women were identified: one whose child was born during the mother’s symptomatic phase (Case 1), and another who suffered a miscarriage (Case 2). From then on, state health surveillance remained alert to possible complications in pregnant women, and four more cases of OROV-positive pregnant women were identified (Cases 3, 4, 5, and 6). These cases were confirmed by laboratory tests (RT-qPCR) after retrospective analysis of individuals with symptoms compatible with arbovirus infection (Figure 2), presenting up to five days of illness and testing negative for DENV, ZIKV, and CHIKV by RT-qPCR.

### 3.2. Case Series

To investigate vertical transmission and fetal loss, six pregnant women and their offspring were analyzed. Figure 1 shows the timeline of the study, while Figure 2 and Table 1 show the mothers’ symptoms and laboratory data from each case. None of the women in these cases had a travel history to previously known endemic areas for OROV.

**Case 1**: A 34-year-old pregnant woman in her sixth week of gestation from Jaqueira Municipality, Pernambuco State, with diabetes and grade 1 obesity, and who had previously tested negative for syphilis, HIV, hepatitis B, hepatitis C, toxoplasmosis, and cytomegalovirus (TORCH panel plus hepatitis and HIV). She presented with fever, severe headache, dizziness, nausea, skin rash, muscle pain, and lower back pain on 6 June 2024 (Figure 2). Five days after symptom onset, her serum was tested as negative for DENV, ZIKV, and CHIKV by RT-qPCR. During follow-up in the eighth week of pregnancy, she experienced a spontaneous miscarriage, but fetal tissues could not be collected for analysis. When retrospective testing for OROV was conducted, RT-qPCR on the serum sample confirmed OROV infection. A second serum sample was collected 24 days after symptom onset in the mother, revealing the presence of IgM antibodies against OROV, while remaining negative for DENV, ZIKV, and CHIKV (Table 2).

**Case 2:** A 32-year-old pregnant woman at 38 weeks of gestation with her first child, from Maraial Municipality, Pernambuco State, with previously negative tests for syphilis, HIV, hepatitis B, hepatitis C, toxoplasmosis, and cytomegalovirus (TORCH panel plus hepatitis and HIV), began experiencing symptoms on 20 May 2024, including fever, severe headache, dizziness, nausea, skin rash, muscle pain, joint pain, and lower back pain (Figure 2). On 22 May 2024 (two days later and still symptomatic), at 38 weeks and 3 days of gestation, she went into spontaneous labor. The newborn, a male without any physical abnormalities, was born weighing 3.376g and measuring 51 cm in length. Three days after birth, the newborn developed a fever lasting for three days. RT-qPCR test results for DENV, ZIKV, and CHIKV were negative. Blood samples from the infant during the febrile period were not collected. After retrospective testing for OROV, the mother’s serum (collected on 22 May 2024) tested positive by RT-qPCR. On 1 July 2024, a second blood sample was collected from both the mother and the child (40 days old), and both tested positive for IgM antibodies against OROV and negative for DENV, ZIKV, and CHIKV (Table 2).

**Case 3:** A 43-year-old pregnant woman, in her 38th week of gestation, from Machados Municipality, Pernambuco State, had previously tested negative for syphilis, HIV, hepatitis B, hepatitis C, toxoplasmosis, and cytomegalovirus (TORCH panel plus hepatitis and HIV). She had no comorbidities, and ultrasound scans (US scans) showed normal fetal development. On 5 August 2024, she attended a routine prenatal check-up, showing an 8.4% weight gain (5.6 kg), normal blood pressure, and fetal growth parameters appropriate for the gestational age (fundal height = 37 cm; fetal heartbeat = 155 bpm) (Table 2).

On 9 August 2024, she presented to the emergency care unit with bilateral mastitis, fever, and chills and reported the loss of fetal movement since the previous night. A few days prior, she had experienced only a mild headache. Clinical evaluation revealed a 2.9 kg weight loss, with blood pressure and other parameters slightly elevated but still within normal limits. However, a decrease in fundal height (34 cm) and the absence of fetal heartbeat and movement were observed. Her serum was collected (9 August 2024) (Table 2). Although Oropouche virus (OROV) genome detection by RT-qPCR was negative, the IgM-ELISA was positive.

**Case 4:** A 21-year-old pregnant woman, in her 29th week of gestation with her first child, from Ipojuca Municipality, Pernambuco State, underwent a gestational ultrasound (US) before the onset of symptoms, which showed normal fetal development without any morphological abnormalities. All TORCHS, hepatitis, and HIV tests were negative. On 15 July 2024, she began experiencing fever, severe headache, and muscle pain (Figure 2). However, one day after the symptoms began, fetal movement ceased, and fetal death was confirmed by US on 16 July 2024. Labor was induced using oxytocin (confirm date of delivery: 18 July 2024), and the fetus was sent for autopsy.

**Figure 3:**
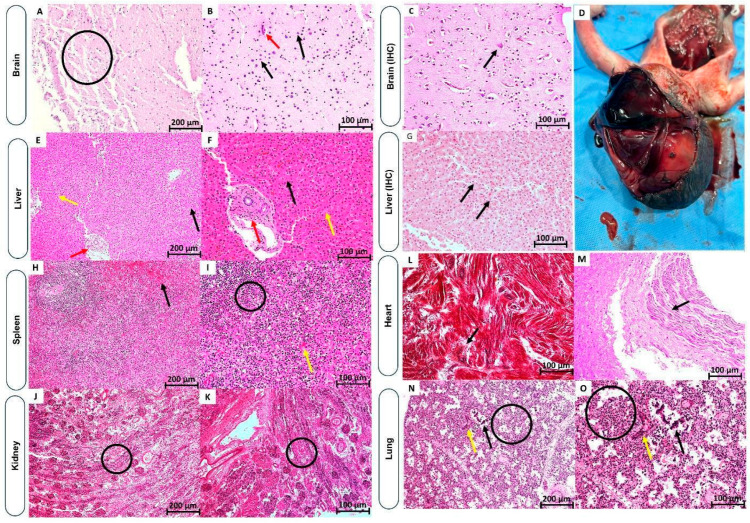
Histopathological, and immunohistochemical (IHC) aspects of Intrauterine Fetal Demise, from case 5, associated with OROV and macroscopic findings from case 3. (**A**,**B**) Brain: Tissue tearing and fragmentation in the brain, accompanied by activated microglia (circle); presence of a neuron with chromatolysis (black arrow) and congested capillary (red arrow); (**C**) nervous tissue displaying OROV antigen labeling by IHC in the cytoplasm of neurons (arrow). (**D**) Fetal necropsy showing epidural hemorrhage in the frontal and occipital regions from case 3. (**E**,**F**) Liver: Necrotic hepatocytes (black arrow). Mild inflammatory infiltrate in the portal area (red arrow). Loss of sinusoidal architecture (yellow arrow). (**G**) Hepatic tissue showing cytoplasmic labeling of hepatocytes (arrows) for the detection of the OROV antigen by IHC. (**H**,**I**) Spleen: Hemorrhagic area (black arrow), reactive pattern (circle), and congestion (yellow arrow). (**J**,**K**) Kidney: Acute tubular necrosis surrounded by mononuclear cell inflammatory infiltrate (circle); inflammatory infiltrate. (**L**,**M**) Heart: Coagulative necrosis with karyolysis in myocardial fibers (black arrow). (**N**,**O**) Lung: Detachment of cylindrical cells in small groups (black arrow); mononuclear cell inflammatory infiltrate (circle) and reactive endothelial cell (yellow arrow).

In maternal serum collected on 18 July 2024 (three days after symptom onset), OROV was detected by RT-qPCR, and the sample was negative for DENV, ZIKV, and CHIKV. Fetal autopsy showed no congenital malformations were observed, consistent with the earlier prenatal US findings. The main finding was severe maceration, including skin desquamation. Autolysis affected all internal organs, although their anatomical structure remained intact. The brain exhibited notable encephalomalacia and epidural hemorrhage in the frontal and occipital regions (Figure 3D). RT-qPCR tests were conducted on fragments of the fetal organs, as well as the placenta and umbilical cord. OROV was detected in the umbilical cord, placenta, brain, kidney, lung, heart, and spleen, while the liver tested negative. The average Ct value was 26.5, with the brain showing the lowest Ct [23], indicating a high viral load.

**Case 5:** This case was reported by PAHO Alert, and a full laboratory investigation was conducted by IEC. The case involved a pregnant woman in the 30th week of gestation, with no travel history, residing in Rio Formoso Municipality, Pernambuco State. Previously, exams showed normal fetal development as well as negative serology for TORCHS, hepatitis, and HIV. On 24 May 2024, the participant reported fever, intense and diffuse headache, rash, and epigastric pain (Figure 2). She disclosed contact with a confirmed OROV case. A lack of fetal movement was noted on 3 June 2024 (10 days after the onset of maternal symptoms). US evaluation performed on 5 June 2024 (10 days after the onset of maternal symptoms), showed absence of a heartbeat (30 weeks and 5 days of gestation), and labor was induced on 6 June 2024. The mother’s serum samples collected on the 9th and 39th days after disease onset showed IgM antibodies for OROV by MAC-ELISA.

**Figure 4:**
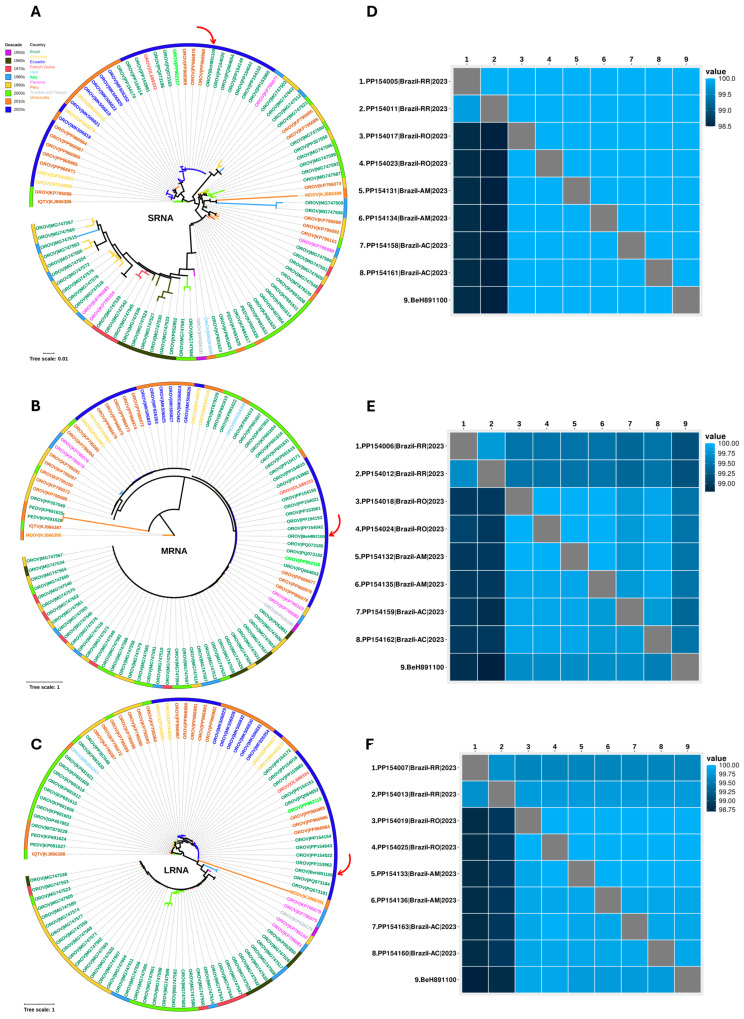
Phylogenetic tree of different OROV strains. Phylogenetic inference was performed using the maximum likelihood (ML) method. (**A**) ML tree based on the nucleotide (nt) sequences of the S-RNA. The TPM3+I+G4 matrix was used as the best nucleotide substitution model. Measurement of the phylogenetic signal in the dataset showed 31.9% unresolved quartets and 68.1% resolved quartets. The numbers at each major node of the tree correspond to bootstrap values in percentages (1000 replicates). (**B**) ML tree based on the nt sequences of the M-RNA. The GTR+F+I+R4 matrix was used as the best nucleotide substitution model. Measurement of the phylogenetic signal in the dataset showed 15.2% unresolved quartets and 84.8% resolved quartets. The numbers at each major node of the tree correspond to bootstrap values in percentages (1000 replicates). The scale bar represents nucleotide divergence per site between sequences. The colors represent different countries. (**C**) ML tree based on the nt sequences of the L-RNA. The GTR+F+I+R2 matrix was used as the best nucleotide substitution model. The measurement of phylogenetic signal in the dataset showed 9.6% unresolved quartets and 90.4% resolved quartets. The numbers at each major node of the tree correspond to bootstrap values in percentage (1000 replicates). The scale bar represents nucleotide divergence per site between sequences. The colors of the sequence IDs represent the countries, and the outer bar represents the decades. The arrow indicates the OROV sequence from fetus BeH891100 (GenBank: S-RNA PQ197204; M-RNA PQ197203; L-RNA PQ197202). Heatmaps showing nucleotide (lower left triangle) and amino acid (upper right triangle) homology identity a matrix between the OROV sequence from the fetus and other OROV sequences from the 2022–2024 outbreak, based on S-RNA (**D**), M-RNA (**E**), and L-RNA (**F**).

Organ fragments from the fetus were collected and tested by RT-qPCR and histopathological analysis. RT-qPCR assays performed on fetal tissues detected OROV in all organs (Table 1). In search of information on co-infection, metagenomic analysis of the virome was performed using a liver sample. NGS only allowed for the sequencing of the OROV genome from the fetal samples, identified as strain BeH891100 (Accession GenBank numbers: PQ197202-PQ197204), with no detection of other viral genomes. The ORFs of S-RNA, M-RNA, and L-RNA were 696 nt, 4.263 nt, and 6753 nt in length, respectively. Phylogenetic analysis of all segments showed that BeH891100 clustered with other strains from Acre, Amazonas, Rondônia, and Roraima states reported during the 2022–2024 OROV outbreak. Despite slight nucleotide differences among the OROV strains from the outbreak (nucleotide identity average: S-RNA = 98.9%, M-RNA = 99.2%, and L-RNA = 99.5%), the amino acid identity for all ORFs remained 100%. Consequently, the OROV reassortment pattern observed in this outbreak is preserved in BeH891100 (Figure 4).

Regarding the fetus, macroscopic analysis of the fetus showed severe maceration, skin desquamation, and a grayish coloration, as well as encephalomalacia. Microscopic analysis revealed endothelial reactivity in the arteries and veins of the umbilical cord, marked by endothelial cell reactivity and loss of vascular integrity. These injuries confirmed the pattern of systemic vasculopathy observed in the fetal organs analyzed. However, the absence of detectable OROV antigens suggests that these changes may be a secondary effect rather than a direct result of viral infection. The placenta was not available for analysis.

Brain tissue showed areas of tissue tearing and fragmentation (Figure 3A). This was accompanied by congested vessels with hypertrophic endothelium associated with an inflammatory infiltrate consisting of lymphocytes, plasma cells, and rare neutrophils surrounding vessels, partially filling the Virchow–Robin spaces (Figure 3B). Reactive endothelium was evident in vessels, often showing altered morphology with enlarged or ruptured endothelial cell junctions, sometimes associated with necrosis and red blood cell extravasation. Additionally, capillary proliferation and congested neovessels with different endothelial cell formats and loose junctions were observed. The interstitial area displayed moderate to severe edema, while activating and proliferating microglia, identified by cells with round and irregular nuclei, surrounded neuronal bodies showing signs of chromatolysis, eosinophilic cytoplasmic condensation, and nuclear pyknosis (Figure 3A). OROV antigen detection by IHC identified neuronal bodies and microglia (Figure 3C), while IHC detection for other arboviruses was negative.

The liver exhibited intense panacinar necrosis, without preferential or specific distribution of lesions in zones I, II, and III. The acinar architecture was completely obliterated, making it impossible to visualize the sinusoidal structure. These lesions primarily consisted of lytic and coagulative necrosis of hepatocytes. The portal tracts showed reactive epithelium in the portal vein and artery, sometimes hypertrophic or exhibiting vascular necrosis. The inflammatory infiltrate was mild and disproportionate to the degree of hepatic parenchymal involvement (Figure 3E,F). IHC revealed OROV antigen detection in the cytoplasm of hepatocytes (Figure 3G).

In the spleen, hyperplasia of the white and red pulp was observed, along with an extensive hemorrhagic area and congested sinusoids, reflecting a reactive pattern (Figure 3H,I), while the kidney showed extensive acute tubular necrosis, associated with a diffuse, mild to moderate interstitial inflammatory infiltrate, and congested glomeruli, interspersed with lymphomononuclear cells (Figure 3J,K). Viral antigen positivity was observed in the walls of the proximal and distal convoluted tubules.

The heart showed myocardial fibers occasionally presenting with coagulative necrosis, characterized by cells undergoing pyknosis, karyorrhexis, and karyolysis. The interstitium displayed mononuclear inflammatory infiltrate and blood vessels with endothelial hypertrophy and endothelial cell necrosis (Figure 3L,M). In the lungs, pneumonitis was observed, characterized by alveolar congestion and a mononuclear infiltrate permeating the alveolar walls. The capillaries presented with reactive, hypertrophic, and congested endothelial cells. Additionally, the respiratory epithelium consisted of ciliated columnar cells, sometimes appearing in small clusters (Figure 3N,O).

**Case 6:** A 28-year-old pregnant woman in the 21st week of gestation with her first child, with no recent travel history, residing in Recife, the capital of Pernambuco State, and with negative serology for TORCHS, hepatitis, and HIV tests. On 14 June 2024, she experienced fever and intense headache, retro-ocular pain, photophobia, rash cutanea, itching, nausea, and vomiting. The symptoms persisted for 5 days (Figure 2). Serial ultrasound examinations conducted before, during, and after the onset of maternal symptoms indicated normal fetal development, with no abnormalities detected in the placenta or umbilical cord. The pregnancy progressed without further complications. On 24 July 2024, during a retrospective investigation, the mother’s serum sample was collected and tested using IgM-ELISA for arboviruses, being positive only for OROV. The newborn, a male, was born on 15 October 2024, via normal vaginal delivery, without complications. Clinically, the newborn appeared healthy, with no physical abnormalities, weighing 3.558 g and measuring 50 cm in length.

## 4. Discussion

Taking a closer look at the history of arbovirus epidemics in Brazil, OROV has been suspected of affecting pregnancy outcomes since the 1980s [43]. Supporting the hypothesis of vertical transmission, a retrospective study conducted on 68 cases of microcephaly with unknown causes in newborns reported in Brazil between 2015 and 2024 confirmed that six cases tested positive for OROV via IgM-ELISA in cerebrospinal fluid (CSF) or serum [9]. Since 2024, further confirmations of OROV’s impact on pregnancy have revealed cases of miscarriage, stillbirth, and microcephaly linked to maternal OROV infection, supported by molecular diagnostics [44,45,46]. Additionally, a case in Cuba provided evidence of potential sexual transmission of OROV [47].

In this case series, we present five possible fetal outcomes resulting from maternal OROV infection during pregnancy: miscarriage, antepartum, stillbirth, IFD, and normal fetal development with no adverse outcomes. We observed a range of maternal symptoms, from oligosymptomatic presentations to classic manifestations, but found no clear relationship between gestational age at the time of infection and fetal outcomes. However, similar to findings for ZIKV infection, the severity of maternal symptoms showed no clear correlation with fetal outcomes, nor did the gestational age at the time of infection [48,49,50].

Case 1 describes a miscarriage resulting from OROV infection, where the pregnant woman contracted the virus during the sixth week of gestation. This case aligns with previous findings from the 1980–1981 OROV outbreak in Amazonas State, Brazil, where two women in their second month of pregnancy also experienced miscarriages [43]. ZIKV also tends to have the greatest impact during the first trimester, often leading to miscarriage as the primary outcome [51,52,53].

Case 2 suggests that OROV infection may trigger antepartum effects, as seen with the onset of spontaneous labor shortly after maternal symptoms appeared at 38 weeks. Studies indicate that viral infections, such as SARS-CoV-2 [54] and ZIKV [55], can predispose pregnant women to preterm labor, raising concerns that OROV could have similar effects. The newborn, delivered without malformations, developed a fever three days later, with OROV infection confirmed via IgM antibodies in his serum. We propose two hypotheses for this infection: vertical transmission during pregnancy or perinatal transmission at delivery; both mechanisms have been documented in ZIKV cases [56].

During the 2022–2024 OROV outbreak, IFD linked to OROV infection was reported for the first time by Brazilian Authority [17] and PAHO [18] base in data from case 5, and latter reinforced by cases 4, and 5. The mechanisms behind IFD in arbovirus infections are complex, involving direct viral damage to placental tissues, immune responses, and vascular complications [57,58].

High viral loads in maternal circulation can facilitate transplacental transmission of viruses to the fetus [59,60,61,62]. Although OROV [13] and ZIKV [63] typically do not exhibit high viremia, in contrast to CHIKV [64], studies have shown that ZIKV can be detected in maternal serum, placental tissue, and fetal organs, correlating with intrauterine fetal demise (IFD) [65]. Similarly, in OROV cases 3, 4, and 5, the viral genome was detected in fetal tissues (Figure 2). The presence of viral RNA in the fetal environment (Table 1) can lead to direct cellular damage, such as necrosis/apoptosis in fetal tissues, or indirectly by cytotoxic immune response, as observed in all tissues in Case 5 (Figure 3), and by IHC in the brain and liver (Figure 3C,G). Maternal clinical symptoms varied across cases and were associated with IFD outcomes. In Case 3, typical symptoms of OROV infection were absent; instead, symptoms resembled mastitis and appeared to be related to the time elapsed since fetal death. The negative RT-qPCR for OROV, along with positive serology, suggested that the viremic phase likely occurred before maternal symptoms emerged. In contrast, Cases 4 and 5 displayed fever, severe headache, and myalgia. Skin rash was noted only in Case 5. Similar to Zika infections [50,53], maternal symptoms in OROV cases also vary widely, indicating that severe fetal outcomes may occur regardless of the presence or absence of classic symptoms associated with the infection.

Phylogenetic analyses indicate that the OROV genome isolated from the IFD case clusters with strains obtained from febrile cases reported in the states of Acre, Amazonas, Rondônia, and Roraima during the 2022–2024 outbreak [12,13] (Figure 4). The genetic divergence among these strains is less than 1% across all segments, indicating a high degree of genomic similarity between the virus from febrile cases and the IFD case. Although in vitro and murine model studies have shown that the OROV strain circulating between 2022 and 2024 exhibits greater viral fitness and virulence compared to the prototype strain isolated in 1960 (BeAn19991) [66], the minimal genetic divergence observed suggests that it is premature to attribute the severity of the IFD case to specific mutations. Further analyses are necessary to evaluate potential associations between viral genetic variation and severe clinical outcomes.

**Figure 5:**
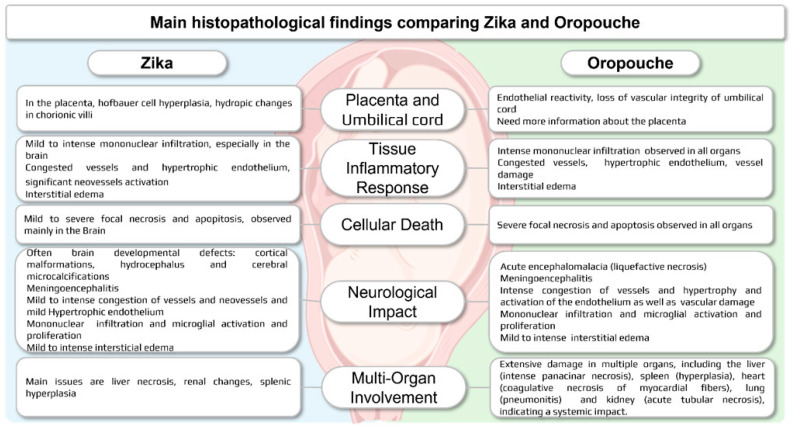
Overview of histopathological findings in fetal tissues infected with OROV [9,70,71] compared to ZIKV [57,63,67,68,69]. Both viruses cause severe CNS damage, characterized by intense inflammatory responses, liquefactive necrosis, and neuronal death (necrosis and/or apoptosis). OROV, however, exhibits intense vascular damage and broader systemic involvement.

Macroscopic brain findings in OROV-infected fetuses reveal severe encephalomalacia (liquefactive necrosis) with extensive tissue tearing and fragmentation, contrasting with the malformations typically seen in ZIKV infections, which often cause microcephaly and cortical malformations. While both viruses lead to significant neurological damage, OROV-induced encephalomalacia is more acute and destructive, with rapid loss of neuronal integrity [9], whereas ZIKV primarily results in brain developmental defects accompanied by dystrophic calcification, suggesting a slower progression of fetal impact [36,44,45,46,47,48,49,50] (Figure 5). Despite the challenges posed by severe fetal maceration and organ autolysis, careful examination combined with ancillary tests [42] can still yield valuable insights into the extent of viral-induced damage. These findings underscore the importance of immune response in the pathogenesis of OROV infection, highlighting its pivotal role in mediating the observed organ damage.

Histopathological findings of OROV infection in human cases of IFD (case 5) and microcephaly [9] share notable similarities with those observed in ZIKV infections [65,67,68,69], particularly in terms of the characteristics of the lesions as well as multi-organ involvement, but OROV infection shows more intense damage. Both viruses are associated with severe CNS injury, characterized by moderate to intense inflammatory responses (microglial activation and mononuclear inflammatory infiltrate), liquefactive necrosis, and neuronal death (either chromatolysis or apoptosis) (Figure 5).

A key feature distinguishing OROV from ZIKV is the observed loss of vascular integrity, particularly in the umbilical arteries and veins, as seen in Case 5, which suggests a profound impact on blood flow. This vascular damage may play a pivotal role in the development of IFD in Cases 3–5 and may contribute to premature labor (Case 2). In the brain, infection with both viruses shows signs of vascular activation and capillary proliferation. However, in OROV-related cases of IFD and microcephaly [9], vascular damage is notably severe, with extensive systemic involvement affecting multiple organs, including the liver, kidneys, and heart. This widespread vascular compromise may also explain the epidural hemorrhage observed in the frontal and occipital regions of the fetus in Case 4 (Figure 3D).

Based on animal models, abundant OROV antigens can be detected in the liver, supporting the idea of systemic viral spread and organ involvement [70,71]. Activation of microglia and Kupffer cells has been noted, suggesting an immune response similar to that observed in human IFD and microcephaly [9] cases. These previous studies have indicated that OROV infection leads to extensive liver damage and cytokine storm in mice lacking key immune signaling pathways. The extent of liver damage during human OROV infection, while severe, appears to be less extensive compared to that caused by yellow fever virus (YFV) [40]. Furthermore, OROV infection leads to significant histopathological changes in the heart (coagulative necrosis of myocardial fibers), lungs (pneumonitis), spleen (hyperplasia), and kidneys (acute tubular necrosis), with patterns of damage that share similarities with other arboviral diseases.

The role of OROV in causing microcephaly has been reported [9], similar to the effects of ZIKV during pregnancy. Both viruses exhibit histopathological similarities, including vascular congestion and inflammatory infiltration by mononuclear cells [63,70,71], although OROV may lead to more severe vascular impairment and systemic lesions. Emerging evidence highlights OROV’s potential for CNS malformations, necessitating further research to compare its effects with those of ZIKV [48,53,63,72,73,74]. Furthermore, in Case 6, although the mother was infected and the fetus developed normally, we cannot ascertain the long-term implications of OROV on the child, underscoring the necessity for ongoing monitoring. This case may illustrate a scenario that could become more prevalent during OROV outbreaks. Therefore, it is essential to closely monitor the broad spectrum of fetal outcomes associated with OROV infection during pregnancy to develop effective public health strategies to address these risks.

## 5. Conclusions

Emerging evidence suggests that OROV may cause CNS malformations and fetal damage, similar to ZIKV, by crossing the placental barrier and leading to fetal malformations, microcephaly, and fetal loss. Our findings, supported by the detection of the OROV genome and antigen in fetal tissues, highlight the potential for vertical transmission and adverse fetal outcomes. While we observed a range of maternal symptoms, there was no clear relationship between gestational age at the time of infection and fetal outcomes. Despite parallels with other arboviruses, OROV’s impact on fetal development remains to be well-characterized, and further research is needed to clarify the incidence of microcephaly and IFD associated with OROV infection. Animal models are essential for understanding disease physiopathologic mechanisms and immune responses, and continued studies are critical for developing public health strategies to mitigate the risks associated with OROV infection during pregnancy.

## Figures and Tables

**Table 1:** Overview of the molecular and serological results of OROV infection in pregnancy.

Test	Samples	Case 1	Case 2	Case 2 Newborn	Case 3	Case 3 Stillborn	Case 4	Case 4 Stillborn	Case 5	Case 5 Stillborn	Case 6	Case 6 Newborn
		Miscarriage	Antepartum		Intrauterine Fetal Demise		Intrauterine Fetal Demise		Intrauterine Fetal Demise		Normal Fetal Development	
RT-qPCR (Ct)	Serum ^1^	34.0	21.0	-	Negative	-	32.0	-	Negative ^¶^	-	-	-
	Umbilical cord blood	-	-	-	-	-	-	27.0	-	-	-	-
	Umbilical cord	-	-	-	-	-	-	37.0	-	25.5	-	-
	Placenta	-	-	-	-	-	-	24.0	-	32.5	-	-
	Brain	-	-	-	-	24.0	-	23.0	-	17.5	-	-
	Liver	-	-	-	-	32.0	-	Negative	-	12.5	-	-
	Spleen	-	-	-	-	30.0	-	24.0	-	12.9	-	-
	Heart	-	-	-	-	29.0	-	27.0	-	17.7	-	-
	Lung	-	-	-	-	Negative	-	28.0	-	18.8	-	-
	Kidney	-	-	-	-	29.0	-	26.0	-	20.5	-	-
ELISA (IgM)	Serum ^2^	Positive	Positive	Positive	Positive	-	-	-	Positive (S1) Positive (S2)	-	Positive	-
	Umbilical cord blood	-	-	-	-	-	-	-	-	Positive	-	-

**Legend:** ^1^—serum collected within five days of symptom onset; ^2^—serum collected after five days of symptom onset; ^¶^—The sample tested negative; however, it was collected beyond the viremic period (>5 days after symptom onset); S1—serum 1 collected 9 days after symptom onset; S2—serum 2 collected 39 days after symptom onset.

**Table 2:** Routine prenatal check-up from case 3, showing maternal–fetal clinical progression. Normal parameters are observed on 5 August 2024, and after 4 days (9 August 2024), fetal demise is confirmed.

Date	Gestational Age	Mother			Fetus			Overview
		Health Condition	Weight	Blood Pressure (mmhg)	Fundal Height	Heartbeat	Fetal Movement	
26 June 2024	31 week, 3 days	Normal	61.1 Kg	100 × 70	32 cm	137 bpm	Present	Normal fetal development
5 August 2024	37 week, 5 days	Normal	66.7 Kg	110 × 70	37 cm	155 bpm	Present	Normal fetal development
9 August 2024 *	38 week, 2 days	Mastitis, Weight loss	63.8 Kg	120 × 80	34 cm	0 bpm	Absent	Confirmed fetal death

* Blood samples collected showed positive results for OROV in the ELISA test but were negative in RT-qPCR.

## Data Availability

The sequences of OROV obtained in this research is openly available at NCBI (https://www.ncbi.nlm.nih.gov/nuccore. Accession GenBank numbers: PQ197202-PQ197204). Any additional information related to this article will be made available by the authors upon request due to sensible patient information.
